# Detection of Human Prion Seeding Activity in Formalin‐Fixed Paraffin‐Embedded Archival Tissues

**DOI:** 10.1111/nan.70028

**Published:** 2025-07-09

**Authors:** Soňa Baranová, Radoslav Matěj, Jiri G. Safar, Karel Holada

**Affiliations:** ^1^ Prion Research Laboratory, Institute of Medical Microbiology, First Faculty of Medicine Charles University Prague Czech Republic; ^2^ Department of Pathology and Molecular Medicine, Third Faculty of Medicine Charles University and Thomayer University Hospital Prague Czech Republic; ^3^ Department of Pathology, First Faculty of Medicine Charles University, and General University Hospital Prague Czech Republic; ^4^ Brain Bank, Third Faculty of Medicine Charles University and Thomayer University Hospital Czech Republic; ^5^ Department of Pathology Case Western Reserve University School of Medicine Cleveland Ohio USA; ^6^ Department of Neurology Case Western Reserve University School of Medicine Cleveland Ohio USA

**Keywords:** FFPE, formalin‐fixed paraffin‐embedded, prion, prion disease, real‐time quaking‐induced conversion assay, RT‐QuIC, SAA, seeding amplification assay

## Abstract

**Aims:**

Formalin‐fixed paraffin‐embedded (FFPE) samples, routinely used in neuropathology, represent an invaluable resource for studying rare diseases like transmissible spongiform encephalopathies (TSE). Despite fixation‐induced protein cross‐linking, prion seeding activity can be effectively detected using the seeding amplification assays. In this study, we employed the second‐generation real‐time quaking‐induced conversion (RT‐QuIC) assay to analyse and quantify human prion seeding activity in FFPE brain tissues.

**Methods:**

FFPE frontal brain tissues were deparaffinised in xylene, followed by rehydration through descending concentrations of ethanol. The prion seeding activity in tissue homogenates was assessed by RT‐QuIC assay utilising short recombinant hamster prion protein (rHaPrP90‐231) as a substrate.

**Results:**

A total of 60 samples, including 30 cases of confirmed TSE, comprising both sporadic and genetic forms, as well as 30 non‐TSE controls, were analysed. Prion seeding activity has been detected in all TSE samples except one sCJD (VV2) and one GSS (P102L) case, corresponding to an assay sensitivity of 93.3%. Conversely, we did not detect any RT‐QuIC positivity in the control group, resulting in 100% specificity. The mean 50% prion seeding dose of FFPE sporadic TSE samples was 10^7.8^/g of brain tissue.

**Conclusion:**

Our study emphasises high sensitivity and specificity of RT‐QuIC assay for prion detection in archival human FFPE brain tissues and demonstrates its diagnostic reliability comparable to other tissue types even after years of storage. The applicability of FFPE samples in RT‐QuIC assays facilitates retrospective diagnostics and provides logistical advantages for sample preservation and transportation.

## SUMMARY

- The RT‐QuIC assay demonstrated high sensitivity and specificity in detecting prion seeding activity in FFPE brain tissues.
- The sensitivity and specificity of the assay were comparable to frozen tissues.
- Prion seeding activity was detectable in FFPE tissues even after years of storage.
- Using FFPE samples facilitates retrospective studies and simplifies sample handling and transport.

## Graphical abstract

The RT‐QuIC assay showed high sensitivity and specificity in detecting prion seeding activity in archival FFPE brain tissues, comparable to results obtained from frozen samples. Notably, prion activity remained detectable in FFPE tissues even after long‐term storage. This highlights the potential of using FFPE samples to support retrospective studies and streamline sample handling and transportation.

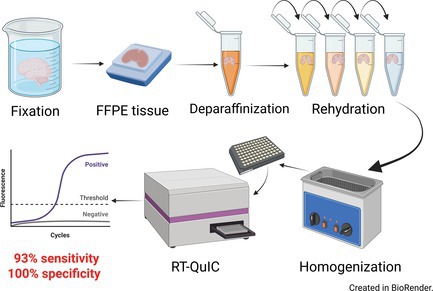

**Abbreviations**
ad
: Alzheimer's disease
AU: arbitrary unit
CJD: Creutzfeldt–Jakob disease
CSF: cerebrospinal fluid
FFPE: formalin‐fixed paraffin‐embedded
FTLD‐tau: frontotemporal lobar degeneration with tau
FTLD‐UPS: frontotemporal lobar degeneration with ubiquitin positive
gCJD: genetic Creutzfeldt‐Jakob disease
GSS: Gerstmann–Sträussler–Scheinker syndrome
ND‐A: nondementia—alcoholism
rHaPrP90‐231: recombinant hamster prion protein, amino acid residues 90‐231
RT‐QuIC: real‐time quaking‐induced conversion
sCJD: sporadic Creutzfeldt–Jakob disease
SD: standard deviation
SD_50_
: seeding dose of 50%
ThT: Thioflavin T
TSE: transmissible spongiform encephalopathy
VPSPr: variable protease‐sensitive prionopathy

## 1 Introduction

Formalin‐fixed paraffin‐embedded (FFPE) tissue samples are widely and routinely used in pathological studies of neurodegenerative disorders owing to their long‐term storage stability and ability to preserve morphological details. However, chemical crosslinking induced by formalin fixation can pose challenges for downstream molecular and genomic analyses.

In prion disease research, the real‐time quaking‐induced conversion (RT‐QuIC) assay has emerged as a highly sensitive (82%–97%) and specific (~100%) technique for detecting prion seeding activity in a variety of samples from patients with transmissible spongiform encephalopathy (TSE) [1]. This assay uses the ability of misfolded prion proteins to template the conversion of recombinant prion protein substrates into amyloid fibrils, which are detected in real‐time using Thioflavin T (ThT) fluorescence. Despite the technical barriers posed by formalin fixation, the infectious conformations of prions demonstrate exceptional heat and organic solvent stability in dry films, and high infectivity can be recovered after rehydration of films [2, 3]. Using these principles, FFPE tissues can be successfully used in RT‐QuIC assays, offering valuable insights into the presence and distribution of prion seeding activity in archived brain samples. Hoover [4] first demonstrated detection and quantification of RT‐QuIC prion seeding activity in FFPE obex samples of white‐tailed deer ( *Odocoileus virginianus* ) with chronic wasting disease. However, since then, most studies have been limited to patients with synucleinopathies. Manne [5] used an RT‐QuIC assay to detect alpha‐synuclein seeding activity in FFPE submandibular gland samples obtained from individuals with confirmed Parkinson's disease and incidental Lewy body disease with 100% specificity and 76% sensitivity. Later, the same group compared alpha‐synuclein seeding activity in frozen (96% sensitivity and 96% specificity) and FFPE (75% sensitivity and 83% specificity) skin tissues in a blinded study [6]. Subsequently, Shin [7] investigated alpha‐synuclein seeding activity in FFPE brain and gastrointestinal tract of patients with Parkinson's disease and multiple system atrophy.

We recently confirmed the efficiency of RT‐QuIC in the analysis of frozen *postmortem* CSF and skin samples from TSE and control patients [8]. In this study, we utilised archived FFPE brain frontal lobe tissues from the same cohort of patients to evaluate the effectiveness of prion detection in archival samples. Prior research has shown that the capability to detect prion seeding activity in archival samples is critical when fresh or frozen tissues are unavailable. Moreover, increasing evidence suggests the common co‐occurrence of other comorbidities such as Alzheimer's disease or primary age‐related tauopathy, which may confound the interpretation of prion‐related pathology and necessitate refined diagnostic approaches. Retrospective analysis of these cases could enhance future diagnostic specificity by clarifying the interplay between these overlapping conditions.

## 2 Materials and Methods

### 2.1 Brain Tissue Samples

All the samples were provided by the Czech National Reference Laboratory for Human Prion Diseases. This study was approved by the ethical committee of the Institute for Clinical and Experimental Medicine and Thomayer University Hospital in Prague (G‐22‐27).

The patient cohort was selected from a retrospective study conducted on *postmortem* cerebrospinal fluid and skin tissues [8]. This supplementary analysis focused on the available archived FFPE tissue samples from the frontal lobe of the brain collected between 2018 and 2020. Sixty autopsy samples were included, comprising 30 cases confirmed to have TSE and 30 control cases with other neurodegenerative conditions (*n* = 25) or other pathologies (*n* = 5). All brains from TSE patients were fixated in buffered 10% formalin for 3–4 weeks, cut into blocks and treated with 96% formic acid according to WHO protocol [9] before embedding into paraffin. In 60% of control samples (*n* = 18), the formic acid treatment was not carried out.

### 2.2 Processing of FFPE Tissue Sections

Proteins were isolated from FFPE sections of the frontal lobe following the protocol described by Hoover [4]. Briefly, ten 4‐μm‐thick wax curls were cut from the paraffin‐embedded tissue blocks. The samples were sequentially washed at RT for 5 min with xylene, followed by ethanol in descending concentrations (100%, 95% and 70%), and finally with PBS (pH 7.4), with each step followed by centrifugation at 15,000 ×*g* for 5 min. The rehydrated brain tissues were weighed (20–60 mg), transferred into a new tube and processed into 10% homogenates in PBS by sonication for 3 × 30s at 40% power (Cup Horn Probe, CPX750 Sonicator, Cole‐Parmer). Tenfold serial dilutions were prepared from 10% brain homogenates, resulting in final end‐point dilutions of brain tissue ranging from 10^−2^ to 10^−8^. Dilutions were prepared in a buffer containing PBS (pH 7.4), 1× N‐2 supplement (Gibco) and 0.1% SDS.

### 2.3 RT‐QuIC Assay

Seeding activity was detected by a second‐generation RT‐QuIC assay as previously described [10, 11, 12]. Briefly, 2 μL of diluted sample was added to 98 μL of reaction buffer containing 11.9 mM phosphate, 1 mM EDTA, 310 mM NaCl (pH 7.4), 10 μM ThT and 0.1 mg/mL recombinant hamster prion protein (rHaPrP90‐231). Samples were incubated in Omega FluoStar reader (BMG, LabTech, Germany) at 55°C for 48 h with repeating 1 min cycles of intermittent shaking at 700 rpm and 1 min rest period. Fluorescence was read every 15 min with excitation at 450 nm and emission at 480 nm, and the gain was set at 2 000.

### 2.4 RT‐QuIC Data Analysis and Quantification of Seeding Activity

The sample was considered positive when the mean maximum of ThT fluorescence from four replicates (wells) exceeded the calculated threshold, and at least two wells displayed elevated ThT fluorescence [1]. The analysis of a sample was repeated when only one of four wells displayed increased fluorescence. The threshold was determined at a 10^−4^ dilution as the average of all maximum ThT fluorescence from negative control samples plus five standard deviations (SD). For the cases with repeated RT‐QuIC, the mean max ThT fluorescence from eight replicates (wells) was included in the threshold calculation.

The results were plotted using GraphPad Prism 5 (GraphPad Software Inc.) and statistically analysed with the nonparametric Mann–Whitney *U* test in SigmaStat (Systat Software Inc.). Difference was considered significant at *p*‐values < 0.05.

For all analysed samples, the seeding dose 50 (SD_50_) was calculated as previously described [13].

## 3 Results

### 3.1 Patient Cohort

FFPE brain tissue samples were obtained from patients with definite TSE (*n* = 30) or controls with other autopsy‐confirmed neurodegenerative disorders or alternative diagnoses (*n* = 30) (Table 1). *Postmortem* interval (PMI) of TSE samples varied between 8 and 96 h (Table S1). All cases included in this analysis were previously characterised in a retrospective study analysing *postmortem* CSF and skin samples [8].

**TABLE 1:** Characterisation of the patient cohort.

	*n*	Mean age ± SD	Male (*n*)/female (*n*)	Mean max. ThT ± SD (AU) ×10^4^	Mean TTT ± SD (h)	Mean AUC ×10^6^	Mean log_10_ SD_50_/g brain
**Sporadic TSEs**	**27**	**65 ± 7**	**14/13**	**12 ± 2.9**	**6.4 ± 2.8**	**3.8**	**7.8**
CJD MM1	11	75 ± 5	9/2	13 ± 1.7	5.6 ± 1.7	4.1	7.9
CJD MM2	2	56, 59	0/2	10; 11	19.5; 7.5	3.3; 3.3	6.7; 7.4
CJD VV1	2	65, 65	0/2	9; 17	4; 7	3.3; 5.9	7.9; 8.4
CJD VV2	4	68 ± 11	2/2	10 ± 5.3	7.5 ± 2.2^a^	3.2	8
CJD MV1	3	70 ± 4	1/2	11 ± 3	7.8 ± 4.7	3.6	7.7
CJD MV2	3	57 ± 12	1/2	12 ± 1.4	7.8 ± 1.7	3.8	7.9
CJD MM1 + 2	1	63	0/1	8.4	4.8	2.5	7.7
CJD VPSPr	1	73	1/0	15	5.8	5	8.4
**Genetic TSEs**	**3**	**70 ± 5**	**1/2**	**9.7 ± 7.1**	**4.3; 5**	**2.8**	**7.4; 8.1**
gCJD E200K	2	65, 75	1/1	14; 13	4.3; 5	3.6; 4.2	7.4; 8.1
GSS P102L	1	69	0/1	1.5	—	0.6	—
**Controls** ^b^	**30**	**72 ± 14**	**18/12**	**2.2 ± 1**	**—**	**0.8**	**—**

*Note:* Detailed demographics, characteristics and RT‐QuIC test results for all tested TSE, including various phenotypes, as well as control non‐TSE formalin‐fixed paraffin‐embedded (FFPE) brain samples.

Abbreviations: AU, arbitrary units; AUC, area under the curve; gCJD, genetic Creutzfeldt‐Jakob disease; GSS, Gerstman‐Sträussler‐Scheinker syndrome; max. ThT, maximal ThT fluorescence; SD, standard deviation; SD_50_, seeding dose of 50%; TSE, transmissible spongiform encephalopathy; TTT, time to threshold.

^a^ The average is calculated from three samples; one sample did not reach the threshold.

^b^ Includes Alzheimer's disease (ad), Dementia with Lewy bodies (DLB), frontotemporal dementia (FTLD), vascular dementia (VaD), synucleinopathy (Syn), nondementia alcoholism (ND‐A), lymphoma infiltration, hypoxic/anoxic brain injury (H/ABI) and encephalitis/diffuse large B‐cell lymphoma (DLBCL).

### 3.2 Assessment of the Prion Seeding Activity in Archive Brain Tissue Samples

**FIGURE 1:**
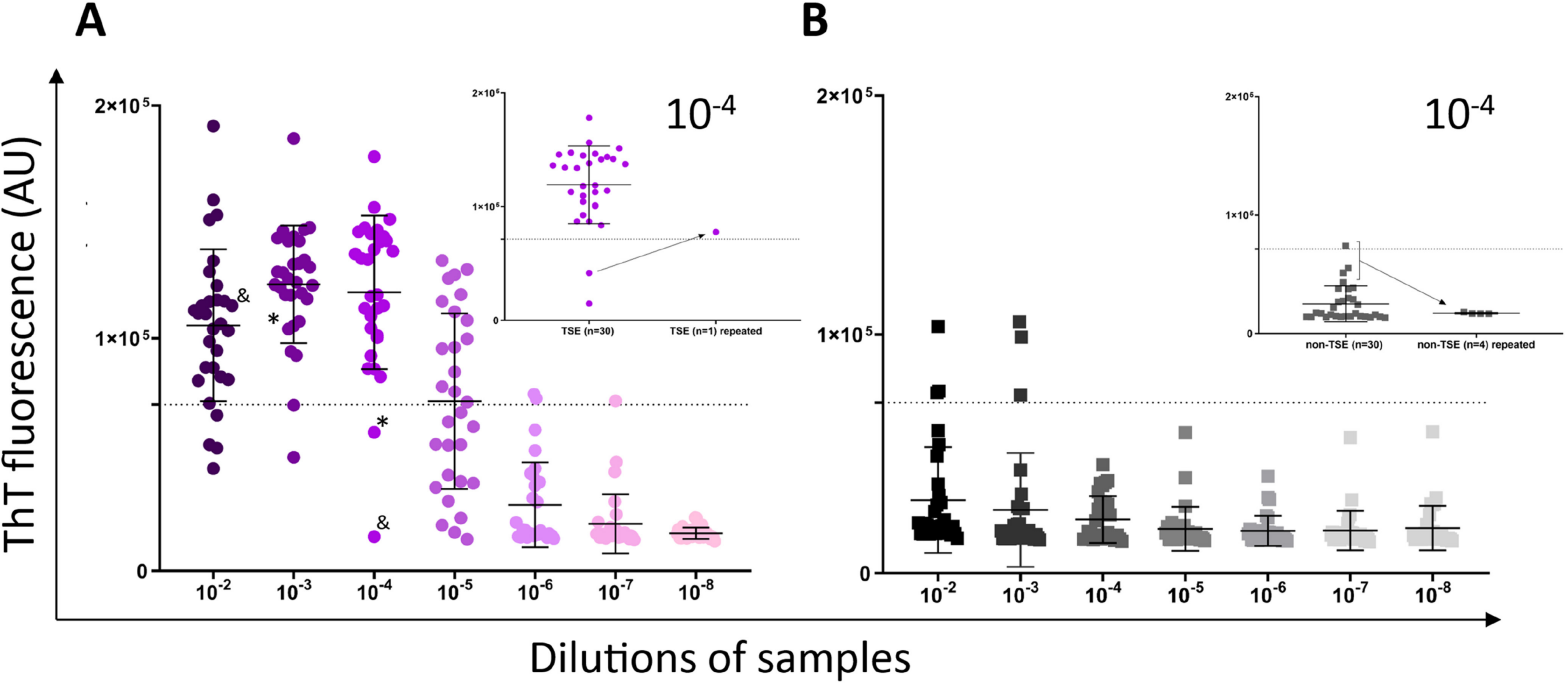
RT‐QuIC detection of prion seeding activity in archived formalin‐fixed paraffin‐embedded (FFPE) brain tissue samples. Dot plots represent the maximum ThT fluorescence for each sample, calculated as the mean maximum ThT intensity from four or eight replicates. The dotted line indicates the threshold for a positive assay outcome. Colour intensity corresponds to increasing dilutions (10^−2^ to 10^−8^) of the analysed samples. Error bars represent mean ± standard deviation (SD). (A) Maximum ThT fluorescence of the TSE samples (*n* = 30). At a 10^−4^ dilution, two samples initially yielded negative results. One TSE VV2 sample showed seeding activity in one well and was reanalysed. Subsequently, the signal increased, as shown in the inset graph (upper left corner). For this case (TSE VV2), the mean of the eight wells was plotted in the graph. (B) Maximum ThT fluorescence of non‐TSE samples (*n* = 30). At a 10^−4^ dilution, four samples exhibited ThT signals above the standard deviation (SD) and were subsequently reanalysed. As shown in the inset graph (upper left corner), the ThT signal significantly decreased following the reanalysis. The mean maximum ThT fluorescence from eight replicates was plotted on the graph.

Brain tissue samples were analysed at end‐point dilutions of 10^−2^–10^−8^, and seeding activity was detected in all TSE samples even when diluted to 10^−5^. However, the ThT signal noticeably decreased at higher dilutions, indicating high variability between patient samples. No TSE sample was positive at a 10^−8^ dilution (Figure 1A). Conversely, some non‐TSE samples exhibited ThT fluorescence signals above the threshold at lower dilutions (10^−2^, 10^−3^), which resulted in the overlap of positive and negative cases. Therefore, all outcomes and parameters of the assay were analysed at a 10^−4^ dilution, where the separation between the positives and negatives was the best.

Of the 30 TSE samples, only two (GSS and VV2) did not reach the calculated threshold (71,405 AU) at a 10^−4^ dilution and were classified as negative. However, it is important to note that these two TSE cases yielded positive RT‐QuIC outcomes at lower dilutions—10^−2^ and 10^−3^ (Figure 1A, asterisks and ampersand). Conversely, four control non‐TSE (FTLD‐UPS, ND‐A, FTLD‐tau and ad) samples at a 10^−4^ dilution gave ThT signals above the SD of the control group. Since the negative TSE VV2 sample and four non‐TSE samples with higher ThT signals all showed elevated fluorescence in only one well, they were reanalysed using the RT‐QuIC assay. After repeated RT‐QuIC analysis, the mean max ThT fluorescence increased for the TSE VV2 sample above the estimated threshold and significantly decreased in all four non‐TSE samples (inset graphs in Figure 1A,B). The mean of the eight wells of the reanalysed samples is plotted in the graph (Figure 1). Therefore, the assay demonstrated 93.3% sensitivity and 100% specificity.

**FIGURE 2:**
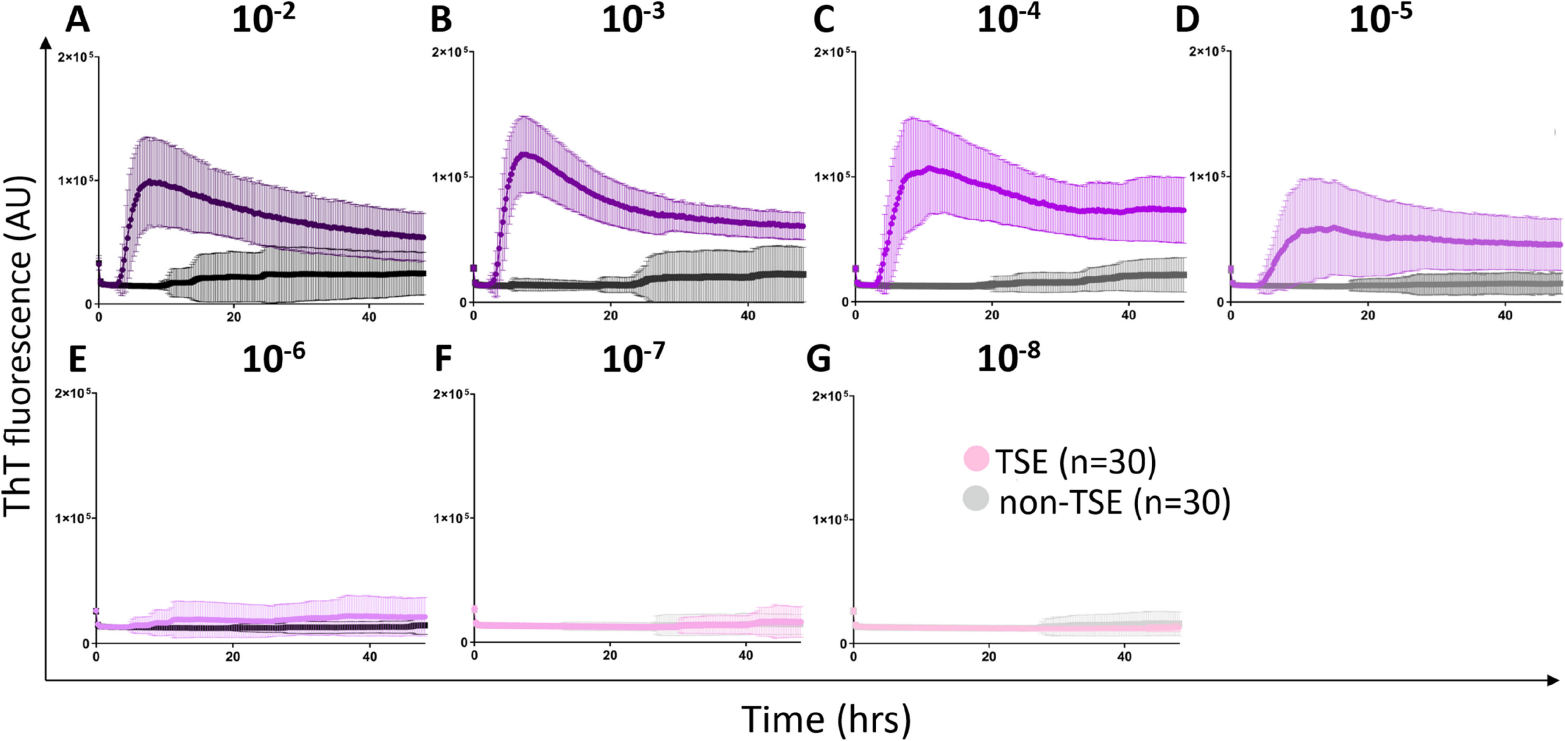
(A–G) RT‐QuIC detection of prion seeding activity in archived formalin‐fixed paraffin‐embedded (FFPE) brain samples. Time course of the mean ThT fluorescence for TSE FFPE samples (*n* = 30, purple shades) and control non‐TSE FFPE samples (*n* = 30, grey shades). The samples were serially diluted to 10^−2^–10^−8^. The points and error bars indicate the mean ThT fluorescence + SD.

The mean max ThT fluorescence for all TSE samples was 12 ± 3.3 × 10^4^. The sample with sporadic TSE VV1 polymorphism exhibited the highest ThT fluorescence signal (17 × 10^4^ AU). In contrast, a sample with sporadic TSE with MM1 + 2 displayed the lowest ThT signal (8.4 × 10^4^ AU). One negative sample with GSS syndrome yielded only 1.5 × 10^4^ AU. For non‐TSE samples, the mean max ThT fluorescence was significantly lower, 2.2 ± 1 × 10^4^ (*p* < 0.0001). The mean time to threshold for TSE samples was 6.3 ± 2.5 h. Interestingly, for two genetic TSE with E200K mutation, the time to threshold was approximately 4.5 h. The mean AUC for TSE samples was 3.7 × 10^6^, while for non‐TSE it was 0.8 × 10^6^ (Figure 2C).

The mean SD_50_ was estimated by limiting the tenfold dilution for all TSE samples except the GSS case, in which prion seeding activity was detected in only two out of four wells and was classified as negative at a 10^−4^ dilution. The SD_50_ varied between sporadic TSE cases, from 10^6.7^/g for MM2 cases to 10^8.4^/g for one VV1 and one VPSPr case (Table 1). The SD_50_ for the two genetic TSE cases with the E200K mutation was 10^7.4^ and 10^8.1^/g.

## 4 Discussion

FFPE samples are essential in research, as they enable long‐term preservation of brain and other tissues affected by rare diseases such as TSE, including Creutzfeldt‐Jakob disease (CJD) or acquired variant form of CJD. FFPE archives provide a valuable resource for retrospective studies, epidemiological research and validation of diagnostic techniques, ultimately contributing to a better understanding of prion diseases and the development of potential therapeutic strategies. Despite the critical role of FFPE samples in the neuropathological evaluation of prion diseases and their potential for retrospective research, no comprehensive RT‐QuIC analysis has been conducted on a cohort of archived patient tissues. Dong [14] compared fixated brains to frozen tissues from 19 patients with MM1, MM2T and MM2C sporadic CJD by testing brain tissue exposure to formalin and formic acid in endpoint RT‐QuIC but not archived FFPE tissues. To the best of our knowledge, this study is the first to retrospectively analyse and quantify human prion seeding activity in archival FFPE samples from a cohort of patients with confirmed sporadic or genetic TSE with different phenotypes using the RT‐QuIC assay.

Using a simple protein extraction protocol and sonication, which was shown to restore seeding activity more effectively [15], we successfully recovered prion seeding activity in all FFPE TSE samples, except for single GSS P102L and CJD VV2 samples. At a 10^−4^ dilution, these two cases did not meet the criteria for RT‐QuIC positivity. However, seeding activity was observed in four wells for GSS P102L at a 10^−2^ dilution and in two wells for CJD VV2 at a 10^−3^ dilution, meeting the criteria for a positive RT‐QuIC outcome. Notably, the GSS P102L case also yielded a negative skin result in our previous retrospective study of CSF and skin samples, whereas the CJD VV2 case was positive in both CSF and skin [8].

Consistent with previous findings [5], RT‐QuIC seeding activity in FFPE samples was detectable at low homogenate dilutions (10^−2^ and 10^−3^). In contrast, in similarly diluted frozen brain samples, seeding activity is quenched presumably by the high concentration of endogenous inhibitors [16]. We assume that this phenomenon is probably caused by washing out of RT‐QuIC inhibitors during xylene and ethanol treatment of FFPE samples. Hoover [16] demonstrated the role of ethanol washes in removing endogenous lipids from brain samples that were responsible for the inhibition of RT‐QuIC assay in lower dilutions. Moreover, previous studies have shown that storage time reduces the quantity of extractable DNA in FFPE tissues, which is known to inhibit RT‐QuIC reactions [17]. However, there is still an ongoing discussion about the exact origin of these inhibitors.

When comparing RT‐QuIC results across different *postmortem* sample types (skin and CSF) from the same patients, we observed distinct trends. In the present study, the average maximum ThT fluorescence of FFPE samples was comparable to that of skin, but significantly lower than that of CSF. Unlike CSF, where the fluorescence signal often reached the upper detection limit of the reader at the same settings, all FFPE samples remained within the measurement range. In contrast to CSF, there was no significant effect of PMI on the RT‐QuIC analysis of FFPE samples. The average SD_50_ in FFPE brain tissues was comparable to the SD_50_ in *postmortem* frozen skin samples. In our retrospective analysis, the mean SD_50_ in frozen brain homogenate samples was approximately three log higher than that in FFPE brain samples [11]. The SD_50_ of FFPE samples decreased compared to that of frozen brain homogenates, presumably because of the combination of crosslinking and formic acid treatment of the prion aggregates and solvent extraction of the samples [2, 3]. Supporting these results, Dong [14] reported a decrease in the SD_50_ value by ~2 log in formalin‐fixed brains (*n* = 19) when compared to frozen tissues. Subsequent formic acid treatment decreased SD_50_ values by another ~3 log, but still, all brain samples remained RT‐QuIC positive which is in accord with our current FFPE data.

On the other hand, we noticed the propensity of some control FFPE samples to produce spontaneous aggregations, mostly at low dilutions and usually just in one well, resulting in a partial overlap of TSE and control non‐TSE RT‐QuIC results. However, this issue can be resolved by the analysis of 10^−4^ diluted samples. Rare spontaneous amyloid formation was also reported by Hoover [4], who tested FFPE obex samples from white‐tailed deer inoculated with chronic wasting disease. Nicholson [18] noted a positive RT‐QuIC reaction in one negative control FFPE sample from a TSE‐free animal. However, it was concluded that this false‐positive result was caused by tissue contamination during the microtomy. In our study, the repetition of RT‐QuIC analysis led to negative results, implying that the previously higher non‐specific ThT signal in the control samples could be caused by stochastic substrate aggregation or may have a technical origin during the setup of the RT‐QuIC assay.

Our study is limited by the small number (*n* = 3) of genetic cases. Two of them gave positive results, and one GSS (P102L) case was classified as negative. Tendency of gCJD (E200K) cases to produce positive RT‐QuIC and the dubious sensitivity of RT‐QuIC in detecting GSS (P102L) cases was noted before [19, 20, 21]. Clearly, more cases of genetic TSE must be analysed before conclusion can be drawn about the robustness of their detection using FFPE brain tissues. Another limiting factor of the study is the occasional occurrence of non‐specific signal in control samples. Although these signals were diminished after repeating the assay, this observation emphasises the importance of numerous controls and a well‐defined threshold for result interpretation.

In summary, we report the high sensitivity (93%) and specificity (100%) of prion seeding activity detection in archival FFPE brain tissues using a simple, well‐established protein extraction method. The achieved sensitivity and specificity were comparable to the published values for other tissues such as CSF and brain homogenates. The application of FFPE samples in RT‐QuIC assays enables retrospective analysis and facilitates the rediagnosis of older cases, where the initial diagnosis remains inconclusive at autopsy or where the disease phenotype is presented atypically. Additionally, FFPE samples offer logistical advantages for long‐distance transportation, enhancing their practical utility in rediagnosis. Furthermore, analysis of available FFPE samples from distinct brain regions may yield novel insights into disease pathogenesis and elucidate the spread patterns of seeds, particularly in cases of TSE complicated by comorbidities.

## Author Contributions

All authors contributed to the study conception and design. Samples and data analysis and manuscript drafting were performed by S.B. Intellectual revisions and manuscript drafting were performed by R.M. and J.G.S. Study concept, interpretation of results, intellectual revisions and manuscript drafting were performed by K.H. The first draft of the manuscript was written by S.B. and all authors commented on previous versions of the manuscript. All authors read and approved the final manuscript.

## Ethics Statement

The study was approved by the Ethics Committee of the Institute for Clinical and Experimental Medicine and the Thomayer University Hospital in Prague, Czech Republic (Approval No. G‐22‐27).

## Consent

The authors have nothing to report.

## Conflicts of Interest

The authors declare no conflicts of interest.

## Supporting information


**Table S1.** Postmortem interval and maximum ThT fluorescence values for every sample with definite transmissible spongiform encephalopathy. Two TSE cases (red with asterisks) were classified as RT‐QuIC negative.

## Data Availability

All RT‐QuIC samples and analyses are available at the Prion Laboratory, Institute of Medical Microbiology, First Faculty of Medicine, Charles University, Prague, Czech Republic. All data will be provided by the corresponding author upon request.
