# Supplementary material for: Detection of Human Prion Seeding Activity in Formalin‐Fixed Paraffin‐Embedded Archival Tissues
Source: Neuropathol Appl Neurobiol. 2025 Jul 9;51(4):e70028. doi: 10.1111/nan.70028 (PMC12239053; doi:10.1111/nan.70028)
Supplement: Supplementary file 1 — Table S1. Postmortem interval and maximum ThT fluorescence values for every sample with definite transmissible spongiform encephalopathy. Two TSE cases (red with asterisks) were classified as RT‐QuIC negative. [file NAN-51-e70028-s001.docx]

## **Supplement**

**Supplementary Table 1**. Postmortem interval and maximum ThT fluorescence values for every sample with definite transmissible spongiform encephalopathy. Two TSE cases (red with asterisks) were classified as RT-QuIC negative.

| **Diagnosis** | **Case no. / year of autopsy** | **PMI (hrs)** | **Max ThT fluorescence (AU) x 10^4^** |
| --- | --- | --- | --- |
| **sTSE**  **CJD (MM1)** | 16/20 | 30 | 15 |
|  | 66/20 | 96 | 12 |
|  | 65/19 | 40 | 10 |
|  | 30/19 | 35 | 14 |
|  | 123/19 | 35 | 14 |
|  | 9/19 | 24 | 10 |
|  | 27/19 | 29 | 15 |
|  | 94/19 | 16 | 13 |
|  | 48/20 | 15 | 14 |
|  | 50/20 | 22 | 14 |
|  | 24/20 | 34 | 12 |
| **sTSE**  **CJD (MM2)** | 33/19 | 30.5 | 11 |
|  | 23/20 | 36 | 10 |
| **sTSE**  **CJD (VV1)** | 56/19 | 17 | 9 |
|  | 66/18 | 55 | 17 |
| **sTSE CJD (VV2)** | 60/20* | 38 | 4 |
|  | 67/20 | 26 | 9 |
|  | 63/19 | 22 | 14 |
|  | 93/19 | 71 | 16 |
| **sTSE**  **CJD (MV1)** | 35/20 | 36 | 11 |
|  | 13/19 | 66 | 9 |
|  | 130/19 | 16.5 | 15 |
| **sTSE**  **CJD (MV2)** | 49/18 | 39 | 14 |
|  | 120/18 | 11 | 11 |
|  | 30/18 | 17 | 11 |
| **sTSE CJD (MM1+2)** | 75/19 | 35 | 8.4 |
| **VPSPr** | 111/18 | 8 | 15 |
| **gTSE**  **gCJD GSS (P102L)** | 52/20* | NA | 1.5 |
| **gTSE gCJD (E200K)** | 85/19 | 43 | 14 |
|  | 14/20 | 47 | 13 |

sTSE – sporadic transmissible spongiform encephalopathy, CJD - Creutzfeldt-Jakob disease, VPSPr – Variably protease-sensitive prionopathy, GSS – Gerstman-Sträussler-Scheinker syndrome, gTSE – genetic transmissible spongiform encephalopathy, PMI – *postmortem* interval, max ThT – maximum Thioflavin T fluorescence, AU – arbitrary units, NA – not available
